# Long-term complete remission of refractory severe idiopathic immune thrombocytopenia (ITP) treated with daratumumab

**DOI:** 10.1007/s00277-022-05035-y

**Published:** 2022-11-17

**Authors:** Tim Strüßmann, Johannes Jung, Jürgen Heinz, Jesus Duque Afonso, Ralph Wäsch, Monika Engelhardt, Justus Duyster, Jürgen Finke, Reinhard Marks

**Affiliations:** Department of Medicine I, Medical Center - University of Freiburg, Faculty of Medicine, University of Freiburg, Hugstetter Straße 55, 79106 Freiburg, Germany

Dear Editor,

Idiopathic immune thrombocytopenia (ITP) is a common acquired autoimmune-mediated thrombocytopenia. Eighty percent of ITP cases are primary with no known underlying medical cause. Generally, primary ITP has a good response to immunosuppressive therapy. First line therapy is glucocorticoids. For relapse or refractory patients, thrombopoetin-receptor-agonists, SYK-inhibitor fostamatinib, or splenectomy are treatment options [1, 2]. As primary ITP is often antibody-mediated, the use of rituximab for selective depletion of CD20-positive B-cells was reported in case reports and studies [3]. However, long-term remissions differ between 10 and 40%. Following B-cell depleting therapies, refractory ITP could be caused due to antibody secretion of long-lived plasma cells [4].

**Fig. 1:**
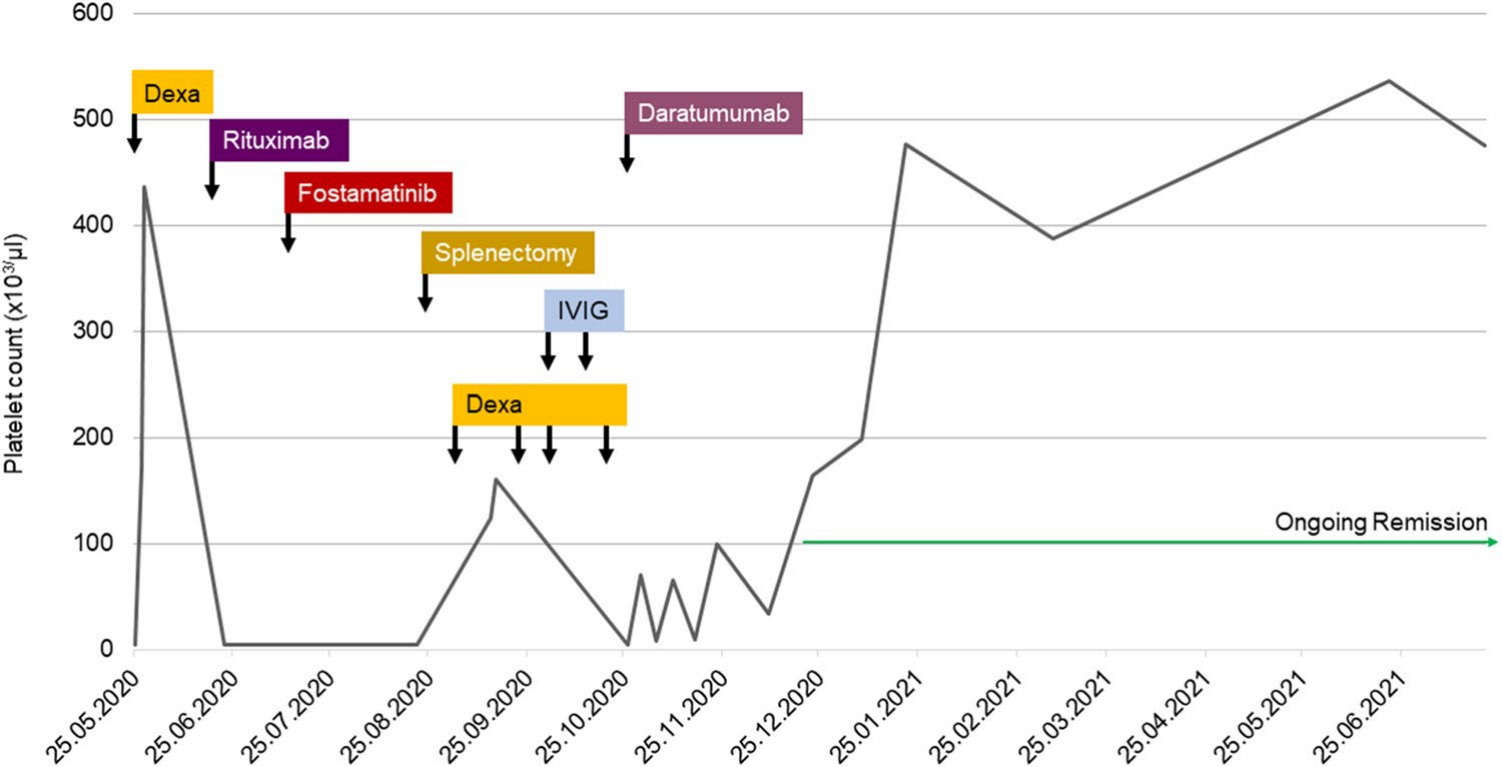
Patients’ course with platelet count (× 10^3^/µL) on the *y*-axis and time (date) on the *x*-axis. Dexa, dexamethasone; IVIG, intravenous immunoglobulin

We hereby report a case of a 39-year old male with a history of childhood idiopathic immune-thrombocytopenia presented at our center with relapsed ITP 25 years after initial splenectomy. The patient relapsed after an infection of the upper respiratory tract. Prior to presentation at our center, the patient received multiple short courses of high-dose steroids, eltrombopag, romiplostime, and triple therapy for *Helicobacter pylori* eradication. The patient presented with petechial bleeding on both legs. Initial platelet count was 4 × 10^9^/L; leukocytes were normal; and hemoglobin was slightly reduced with 12.1 g/dL platelet count in citrate plasma was 2 × 10 10^9^/L. Howell-Jolly-bodies could not be detected, while a MRI of the abdomen showed three spleen regenerates. We initiated rituximab (375 mg/m^2^) weekly for 8 weeks and hereafter therapy was switched to fostamatinib; however, both did not result in an increase in platelets. A laparoscopic splenectomy of spleen regenerates was performed but did not change peripheral platelet counts. At this time point, the obtained bone-marrow biopsy showed an increased megakaryopoiesis with no hints of a hematologic malignancy. During the next months, only repetitive dexamethasone and IVIG could recover platelet counts for short time periods. Additionally, repetitive steroid therapy led to Cushing Syndrome and CMV-reactivation. With this refractory history of ITP, we initiated daratumumab 1800 mg s.c. on days 1, 8, 15, and 22 q29 for three cycles after patient consent to off-label use. Six weeks after onset of daratumumab, platelet count recovered and remained in continuous remission for now 20 months (Fig. 1). Signs and symptoms of Cushing Syndrome rapidly vanished. We obtained patients’ informed consent to data collection and reporting.

Daratumumab is widely used in multiple myeloma today, targeting CD38 on neoplastic plasma cells [5]. First evidence of efficacy of daratumab in immune cytopenias was gained in patients with post-transplant related autoimmune hemolytic anemia and immune cytopenias [6, 7]. Moreover, daratumumab seems effective in children and young adults with autoimmune cytopenias [7]. Migdady et al. reported one case of successful treatment of thrombocytopenia with daratumumab after allogeneic transplant and contribute a literature review of 7 patients successfully treated with daratumumab in the posttransplant setting [8]. Crickx and colleagues described five cases of refractory ITP, whereby the number of daratumumab i.v.-infusions varied between 4 and 7, and 3 out of 5 patients responded well with a treatment response at the time of the report of 3 and 12 months, respectively [9]. Reported patients with immune thrombocytopenia and daratumumab treatment are summarized in Table 1. To conclude, daratumumab seems a valid off-label treatment option for refractory ITP patients. In our patient, s.c.-daratumumab administration was safe and resulted in a long-term remission, leading us in two recent severely refractory ITP patients to conventional ITP treatment to treat them likewise. Of note, one patient responded like our first patient, but the other patient unfortunately did not respond to daratumumab treatment.

**Table 1:** Reported patients with immune cytopenias and daratumumab treatment (patients in the posttransplant-setting are not listed)

Source paper	# of pts	Condition	Daratumumab treatment	Outcome
Crickx et al. [9]	5	ITP	4–7 daratumumab infusions	Complete remission in 3/5 pts. (with a duration of response from 3–12 months)
	1	AGS	4 daratumumab infusions	Treatment failure
	2	Warm AIHA	4 and 11 daratumumab infusions	1 complete remission, 1 treatment failure
Jain et al. [10]	1	Warm AIHA	4 daratumumab infusions	Steroid-free period of 5 months
Rieger et al. [11]	3	Warm AIHA	1 pt. with 3 and 2 pts. with 6 daratumumab applications	1 patient with disease control (with 16-months of follow-up), 2 pts relapsed (after 2 and 5 months)
	1	Warm AIHA + ITP	6 daratumumab applications	Disease control with a follow-up of 15 months
Strüßmann et al.	1	ITP	12 daratumumab s.c. applications	Complete remission with a follow-up of 20 months

*AGS* acquired Glanzmann syndrome, *AIHA* autoimmune hemolytic anemia
